# The complete chloroplast genome sequence of *Zehneria japonica* (Thunb.) H. Y. Liu (Cucurbitaceae), a medicinal plant

**DOI:** 10.1080/23802359.2025.2571717

**Published:** 2025-10-13

**Authors:** Hengyuan Hu, Menghao Wang, Yan Zhuang, Lianzhen Li, Xiaoming Guo, Peiyan Ai, Jianfeng Chang, Yanan Cao, Junjie Wei (Jackie Ngai)

**Affiliations:** a College of Agronomy, Henan Agricultural University, Zhengzhou, China; b College of Plant Protection, Henan Agricultural University, Zhengzhou, China; c College of Life Sciences, Henan Agricultural University, Zhengzhou, China

**Keywords:** *Zehneria japonica*, chloroplast genome, phylogenetic analysis

## Abstract

*Zehneria japonica* (Thunb.) H. Y. Liu is a traditional Chinese medicinal plant. We sequenced and annotated its complete chloroplast genome, which is 157,052 bp in length with 36.99% GC content. It exhibits a typical quadripartite structure, including a large single-copy region of 86,849 bp, a small single-copy region of 17,867 bp, and two inverted repeat regions of 26,168 bp. The genome encodes 131 genes (86 protein-coding, 37 tRNA, and 8 rRNA). Phylogenetic analysis based on chloroplast genomes supports the monophyly of *Zehneria* species. These results enrich genomic resources and provide a foundation for future phylogenetic and evolutionary studies within *Zehneria*.

## Introduction

*Zehneria japonica* (Thunb.) H. Y. Liu 1989, an annual scandent herb in the family Cucurbitaceae, is mainly distributed in China, Japan, Korea, Vietnam, Indian Peninsula, Indonesia (Java), and the Philippines, and often in the shade and wet places, roadsides, field edges, and thickets (Wu et al. 2011). Its tuberous roots or whole herb are used as traditional Chinese medicine for urinary incontinence, eczema of the skin, bleeding from traumatic injuries, and venomous snake bites (Ye et al. 2021). It is also found to have angio-suppressive activity (Roldan et al. 2018).

According to *Flora of China* (Wu et al. 2011), there are about 55 plant species in the genus *Zehneria* around the world, and four species in China. Deficiency on genetic information of *Zehneria*, especially genomic sequence, limits the research on the genus. Until now, only eight chloroplast genomes of the *Zehneria* are available in the NCBI (National Center for Biotechnology Information, https://www.ncbi.nlm.nih.gov/) database, including seven known *Zehneria* species and one unknown species; however, the chloroplast genome of *Z. japonica* is not included. Here, we assembled and annotated the chloroplast genome of *Z. japonica*, and investigated its phylogenetic status.

## Materials and methods

**Figure 1.**
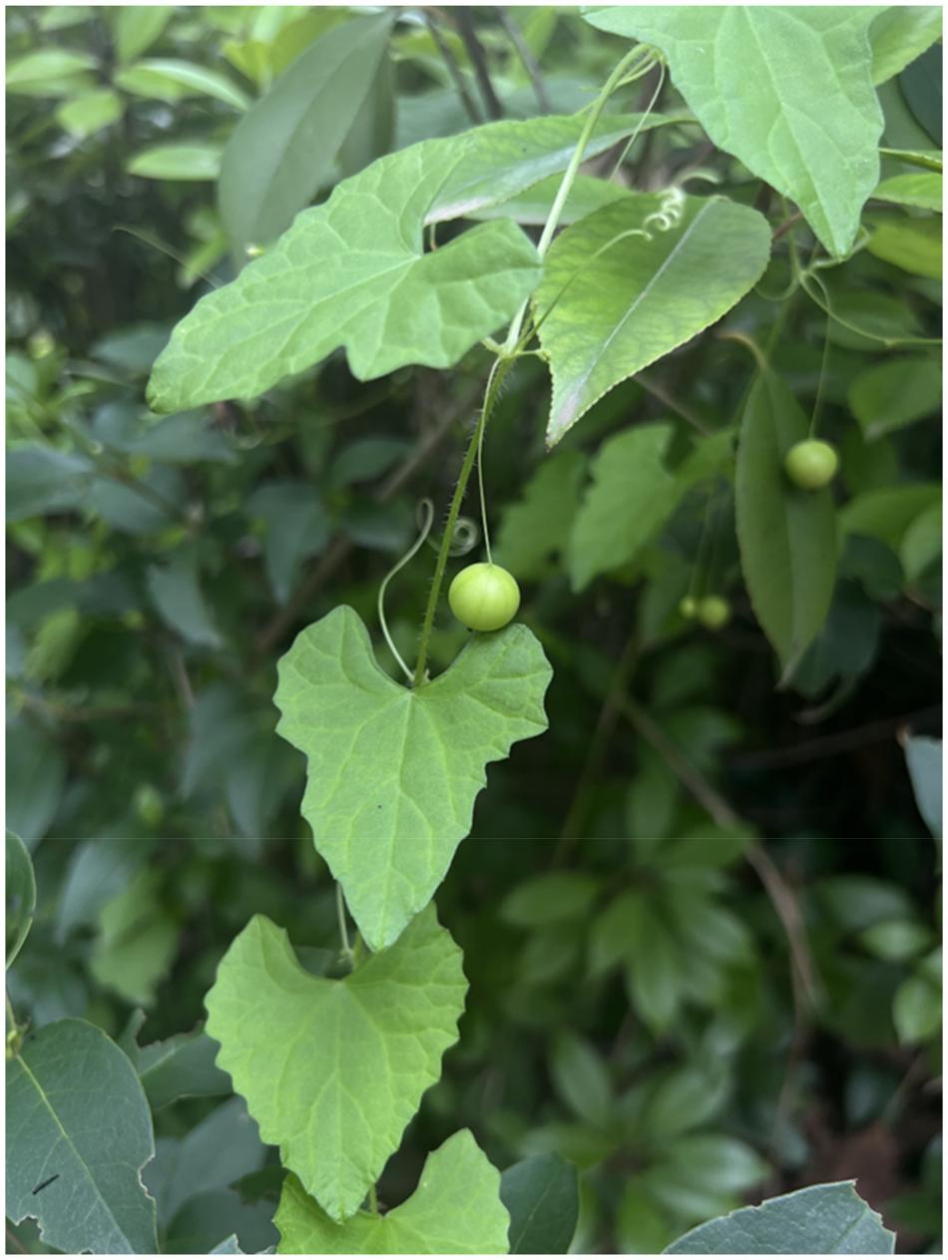
Photograph of *Zehneria japonica.* Photograph by Junjie Wei. *Z. japonica* is an annual scandent herb with triangular-ovate or ovate-cordate leaf blade and slender petiole. The plant is monoecious with small male and female flowers, nearly spherical fruit.

Fresh and healthy leaves were collected from the medicinal botanical garden of Henan Agricultural University, Longzi Lake Campus, Zhengzhou, Henan, China (34.7970°N, 113.8233°E). The plant was identified by Prof. Lianzhen Li as *Zehneria japonica* (Thunb.) H. Y. Liu, and the voucher specimen was deposited in the Herbarium of College of Agronomy, Henan Agricultural University (voucher number: JN20240901001, Junjie Wei, JunjieNgai2020@henau.edu.cn, see Figure 1).

Whole genomic DNA (gDNA) was extracted using CTAB method (Doyle and Doyle 1987). Subsequently, the isolated gDNA was delivered to Bena Technology Co. Ltd. (Wuhan, China) for DNA library construction and high throughput sequencing with pair-end (PE150) reads via the DNBSEQ-T7 platform. The chloroplast gDNA was assembled by GetOrganelle v1.7.7.1 (Jin et al. 2020) and visualized by Bandage v0.8.1 (Wick et al. 2015), then was annotated by PGA (Qu et al. 2019) with the chloroplast genome sequence of *Z. longiflora* (NCBI accession PP831691.1) as a reference. The annotated genome was manually modified and corrected using Geneious Prime v2025.0.2 (Kearse et al. 2012). The circular chloroplast genomic map was built by OGDraw (Greiner et al., 2019).

To clarify the phylogenetic relationships among *Z. japonica* and other 22 related species in Benincaseae (Cucurbitaceae), the complete chloroplast genome sequences with only one copy inverted repeat (IR) region were aligned via MAFFT v7.505 (Katoh et al. 2002) using ‘–auto’ strategy and normal alignment mode. The direction of the SSC region in each genome sequence was checked and manually corrected to ensure consistency with that of *Z. japonica*. The phylogenetic tree was constructed via IQTree tool within PhyloSuite v1.2.2 (Zhang et al. 2020) with the maximum-likelihood (ML) method and 1000 bootstrap values. The chloroplast genome of *Hemsleya amabilis* belonging to Gomphogyneae (Cucurbitaceae) was chosen as outgroup.

## Results

**Figure 2.**
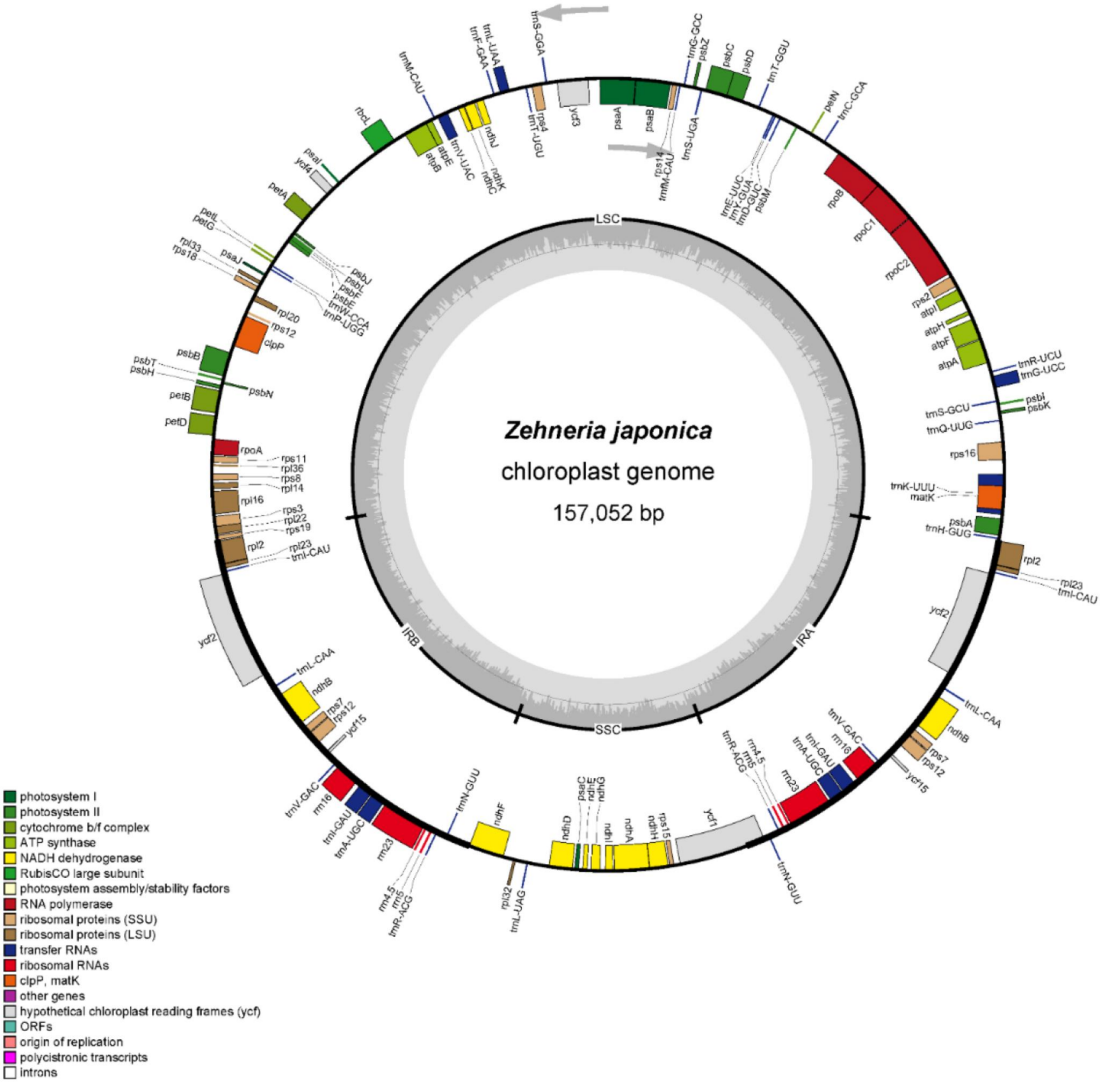
The complete chloroplast genome map of *Zehneria japonica.* The outer circle shows distribution of genes. Genes inside the circle are transcribed clockwise while those on the outside are transcribed counterclockwise. Genes are colored differently according to their role. In the inner circle, GC content is displayed in a deeper shade of gray, while the AT content is displayed in a lighter shade.

The chloroplast genome of *Z. japonica* (GenBank accession: PV819072.1) spans 157,052 base pairs (bp) in total length, with a typical quadripartite structure consisting of two IR regions (IRs, each 26,168 bp), a large single-copy region (LSC, 86,849 bp), and a small single-copy region (SSC, 17,867 bp). The overall GC content of the genome is 36.99%, with the IRs, LSC, and SSC exhibiting GC contents of 42.75%, 34.74%, and 31.09%, respectively. In terms of sequencing coverage, the assembled genome shows a minimum read mapping depth of 410×, a maximum of 13,291×, and an average of 4705× (see Figure S1). A total of 131 genes are annotated in the chloroplast genome, including 86 protein-coding genes, 37 transfer RNA (tRNA) genes, and eight ribosomal RNA (rRNA) genes (Figure 2). Among these, 18 genes are present in duplicate copies, including seven protein-coding genes (*ndhB*, *rpl2*, *rpl23*, *rps7*, *rps12*, *ycf2*, *ycf15*), seven tRNA genes (*trnA-UGC*, *trnI-CAU*, *trnI-GAU*, *trnL-CAA*, *trnN-GUU*, *trnR-ACG*, *trnV-GAC*), and all four rRNA genes (*rrn5*, *rrn16*, *rrn4.5*, *rrn23*). Intron distribution analysis reveals that three genes (*clpP*, *rps12*, *ycf3*) contain two introns each, while nine protein-coding genes (*ndhA*, *ndhB*, *petB*, *petD*, *atpF*, *rpl2*, *rpl16*, *rps16*, *rpoC1*) and six tRNA genes (*trnA-UGC*, *trnG-UCC*, *trnI-GAU*, *trnK-UUU*, *trnL-UAA*, *trnV-UAC*) harbor one intron each. Additionally, the *Z. japonica* chloroplast genome includes 11 cis-splicing genes (*clpP*, *ycf3*, *atpF*, *ndhA*, *ndhB*, *petB*, *petD*, *rpl16*, *rpl2*, *rpoC1*, *rps16*; Figure S2) and one trans-splicing gene, *rps12* (Figure S3).

**Figure 3.**
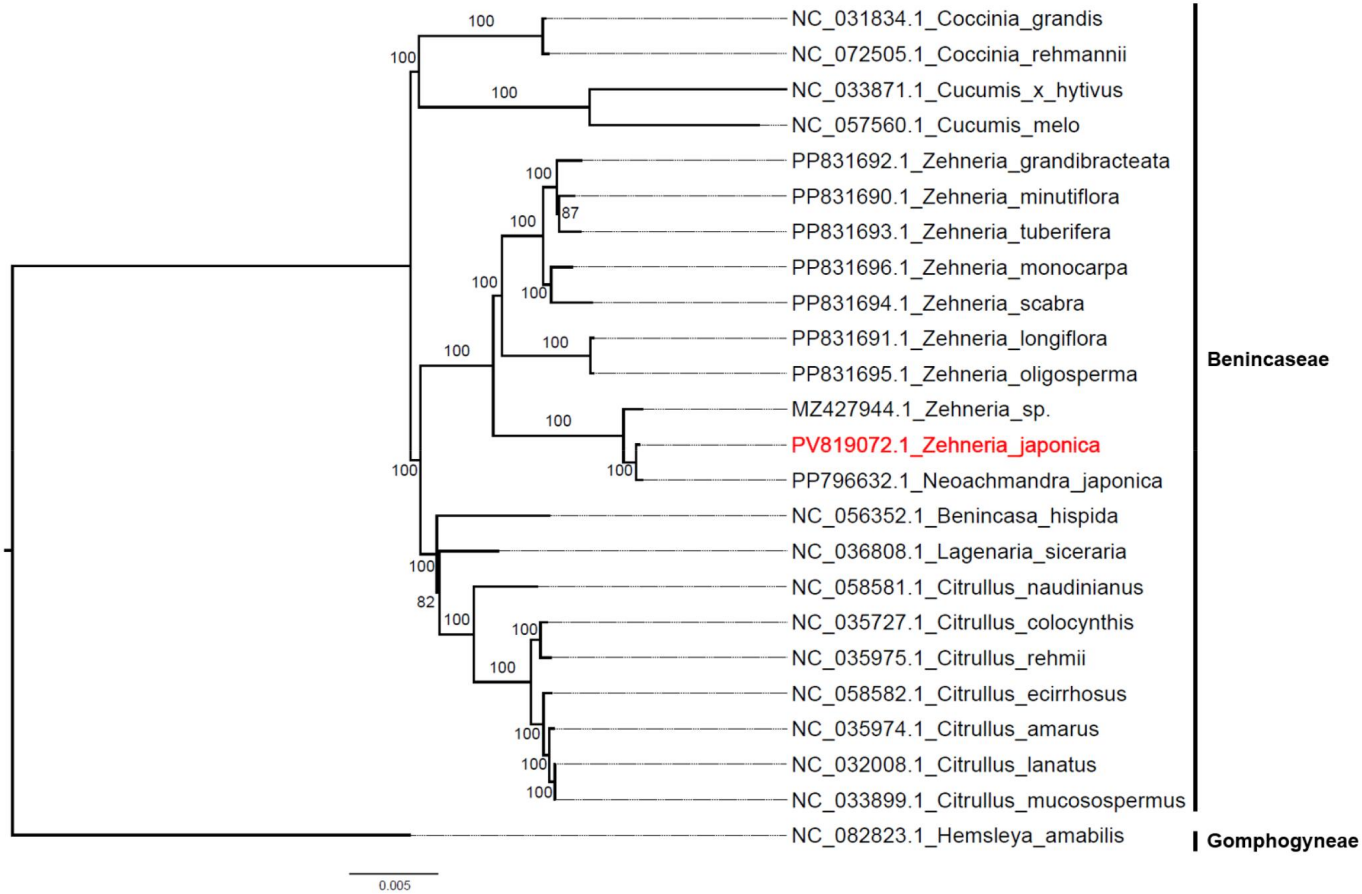
Phylogenetic position of *Zehneria japonica* in Benincaseae (Cucurbitaceae). The tree with aligned tip labels (dash-dotted guide lines) was constructed with maximum-likelihood (ML) method and bootstrap replicate was set as 1000, numbers above branches represent bootstrap value. *Hemsleya amabilis* belonging to Gomphogyneae (Cucurbitaceae) was selected as outgroup. The target genome of *Z. japonica* was in red. The following sequences were used: *Z. minutiflora* (PP831690.1) *Z. longiflora* (PP831691.1), *Z. grandibracteata* (PP831692.1), *Z. tuberifera* (PP831693.1), *Z. scabra* (PP831694.1), *Z. oligosperma* (PP831695.1), *Z. monocarpa* (PP831696.1), *Zehneria* sp. (MZ427944.1), *Neoachmandra japonica* (PP796632.1), *Lagenaria siceraria* (NC_036808.1), *Citrullus amarus* (NC_035974.1), *Citrullus colocynthis* (NC_035727.1, Zhu et al. 2017), *Citrullus ecirrhosus* (NC_058582.1), *Citrullus lanatus* (NC_032008.1, Zhu et al. 2016), *Citrullus mucosospermus* (NC_033899.1), *Citrullus naudinianus* (NC_058581.1), *Citrullus rehmii* (NC_035975.1), *Coccinia grandis* (NC_031834.1), *Coccinia rehmannii* (NC_072505.1), *Cucumis* × *hytivus* (NC_033871.1), *Cucumis melo* (NC_057560.1), *Benincasa hispida* (NC_056352.1), and *H. amabilis* (NC_082823.1).

To investigate the phylogenetic relationships of *Z. japonica* with other plants in Benincaseae (Cucurbitaceae), a phylogenetic tree (Figure 3) was constructed using complete chloroplast genomes. The results showed that all species of Benincaseae formed a single clade. Within the genus *Zehneria*, species clustered together with high bootstrap support values, suggesting that the genus is monophyletic. Moreover, two distinct clades were observed within *Zehneria*. One clade included *Z. japonica*, *Neoachmandra japonica* (GenBank accession: PP796632.1; a synonym of *Z. japonica*), and an unidentified *Zehneria* species (GenBank accession: MZ427944.1). The other clade consisted of seven recognized *Zehneria* species, indicating that *Z. japonica* is somewhat distinct from the other members of the genus. In addition, species of the genera *Citrullus*, *Lagenaria*, and *Benincasa* clustered together as a sister clade to *Zehneria*, demonstrating a close relationship between these genera and *Zehneria*.

## Discussion and conclusions

Here, we report the complete chloroplast genome sequence of *Zehneria japonica*, which exhibits a typical circular quadripartite structure consistent with most angiosperm chloroplast genomes (Jansen et al. 2005; Jansen and Ruhlman 2012). It should be noted that this species is classified as *Neoachmandra japonica* (Thunb.) W.J. de Wilde & Duyfjes, 2006 in NCBI taxonomy (https://www.ncbi.nlm.nih.gov/), which is regarded as a synonym of *Z. japonica* in POWO (Plants of the World Online, https://powo.science.kew.org/), GBIF (Global Biodiversity Information Facility, https://www.gbif.org/), and WFO (World Flora Online, https://www.worldfloraonline.org/). *Neoachmandra* is also believed to a genus segregated from *Zehneria* (Dwivedi et al. 2018). The chloroplast genome of *N. japonica* (GenBank accession: PP796632.1), recently released through direct submission, shows a length of 157,102 bp. Notably, the SSC region orientation in PP796632.1 differs from that of model plants such as *Arabidopsis thaliana* (GenBank accession: NC_000932.1) and *Nicotiana tabacum* (GenBank accession: NC_001879.2; see Figure S4), which may potentially cause confusion in future studies.

Comparative analysis reveals that the gene content of *Z. japonica* chloroplast genome is highly conserved with other published *Zehneria* species (including PP796632.1), enabling robust phylogenetic investigations within this genus. Our phylogenetic reconstruction demonstrates that *Zehneria* (including *N. japonica*) forms a well-supported monophyletic group comprising two distinct clades (Figure 3), corroborating previous findings by Dwivedi et al. (2018) based on ITS region sequences and combined nrDNA (ITS) and cpDNA (*trnL* intron, *trnL*-*trnF* spacer, *rpl20*-*rps12*, *matK*) analyses. Significantly, *N. japonica* and *Z. japonica* cluster together with strong bootstrap support. Using *H. amabilis* (Gomphogyneae, Cucurbitaceae) as the outgroup and including representatives from other Benincaseae genera, our phylogenetic analysis further confirms Benincaseae as a monophyletic group (Renner and Schaefer 2016; Zhu et al. 2017; Song et al. 2022). The complete chloroplast genome sequence of *Z. japonica* presented in this study could facilitate not only precise determination of the evolutionary position of *Z. japonica* within Cucurbitaceae but also development of species-specific molecular markers.

## Supplementary Material

Supplementary_material.pdf

## Data Availability

The genome sequence data that support the findings of this study are openly available in GenBank of NCBI at https://www.ncbi.nlm.nih.gov/ under accession no. PV819072.1. The associated BioProject, Bio-Sample, and SRA numbers are PRJNA1292402, SAMN49999779, and SRR34564514, respectively.

## References

[CIT0001] Doyle JJ, Doyle JL. 1987. A rapid DNA isolation procedure for small quantities of fresh leaf tissue. Phytochem Bull. 19(1):11–15.

[CIT0002] Dwivedi MD, Barfield S, Pandey AK, Schaefer H. 2018. Phylogeny of *Zehneria* (Cucurbitaceae) with special focus on Asia. Taxon. 67(1):55–65. 10.12705/671.4

[CIT50123197] Greiner S, Lehwark P, Bock R. 2019. OrganellarGenomeDRAW (OGDRAW) version 1.3.1: expanded toolkit for the graphical visualization of organellar genomes. Nucleic Acids Res. 47(W1):W59–W64. 10.1093/nar/gkz238. 30949694 PMC6602502

[CIT0003] Jansen RK, Raubeson LA, Boore JL, DePamphilis CW, Chumley TW, Haberle RC, Wyman SK, Alverson AJ, Peery R, Herman SJ. 2005. Methods for obtaining and analyzing whole chloroplast genome sequences. In: Zimmer EA, Roalson EH, editors. Methods in enzymology. Vol. 395. London: Academic Press. 10.1016/S0076-6879(05)95020-9 15865976

[CIT0004] Jansen RK, Ruhlman TA. 2012. Plastid genomes of seed plants. In: Bock R, Knoop V, editors. Genomics of chloroplasts and mitochondria. Advances in photosynthesis and respiration. Vol. 35. Dordrecht: Springer.

[CIT0005] Jin J, Yu W, Yang J, Song Y, DePamphilis CW, Yi T, Li D. 2020. GetOrganelle: a fast and versatile toolkit for accurate *de novo* assembly of organelle genomes. Genome Biol. 21(1):241. 10.1186/s13059-020-02154-5 32912315 PMC7488116

[CIT0006] Katoh K, Misawa K, Kuma K, Miyata T. 2002. MAFFT: a novel method for rapid multiple sequence alignment based on fast Fourier transform. Nucleic Acids Res. 30(14):3059–3066. 10.1093/nar/gkf436 12136088 PMC135756

[CIT0007] Kearse M, Moir R, Wilson A, Stones-Havas S, Cheung M, Sturrock S, Buxton S, Cooper A, Markowitz S, Duran C. 2012. Geneious Basic: an integrated and extendable desktop software platform for the organization and analysis of sequence data. Bioinformatics. 28(12):1647–1649. 10.1093/bioinformatics/bts199 22543367 PMC3371832

[CIT0008] Qu X, Moore MJ, Li D, Yi T. 2019. PGA: a software package for rapid, accurate, and flexible batch annotation of plastomes. Plant Methods. 15(1):50. 10.1186/s13007-019-0435-7 31139240 PMC6528300

[CIT0009] Renner SS, Schaefer H. 2016. Phylogeny and evolution of the Cucurbitaceae. In: Grumet R, Katzir N, Garcia-Mas J, editors. Genetics and genomics of Cucurbitaceae. Plant genetics and genomics: crops and models. Vol. 20. Cham: Springer.

[CIT0010] Roldan MJ, Chin T, Tsai Y, Castillo A, Villaflores OB. 2018. Cytotoxic and angiosuppressive potentials of *Zehneria japonica* (Thunb. ex Murray) SK Chen (Cucurbitaceae) crude leaf extracts. Philippine J Health Res Dev. 22(2):43–52.

[CIT0011] Song W, Chen Z, He L, Feng Q, Zhang H, Du G, Shi C, Wang S. 2022. Comparative chloroplast genome analysis of wax gourd (*Benincasa hispida*) with three Benincaseae species, revealing evolutionary dynamic patterns and phylogenetic implications. Genes. 13(3):461. 10.3390/genes13030461 35328015 PMC8954987

[CIT0012] Wick RR, Schultz MB, Zobel J, Holt KE. 2015. Bandage: interactive visualization of *de novo* genome assemblies. Bioinformatics. 31(20):3350–3352. 10.1093/bioinformatics/btv383 26099265 PMC4595904

[CIT0013] Wu Z, Raven PH, Hong D, editors. 2011. Flora of China. Vol. 19. Beijing: Science Press & St. Louis: Missouri Botanical Garden Press.

[CIT0014] Ye H, Li C, Ye W, Zeng F, Liu F, Liu Y, Wang F, Ye Y, Fu L, Li J. 2021. Medicinal angiosperms of Flacourtiaceae, Tamaricaceae, Passifloraceae, and Cucurbitaceae. In: Ye H, Li C, Ye W, Zeng F, editors. Common Chinese Materia Medica. Vol. 3. Singapore: Springer.

[CIT0015] Zhang D, Gao F, Jakovlić I, Zou H, Zhang J, Li WX, Wang GT. 2020. PhyloSuite: an integrated and scalable desktop platform for streamlined molecular sequence data management and evolutionary phylogenetics studies. Mol Ecol Resour. 20(1):348–355. 10.1111/1755-0998.13096 31599058

[CIT0016] Zhu Q, Cui H, Zhao Y, Gao P, Liu S, Wang P, Luan F. 2016. The complete chloroplast genome sequence of the *Citrullus lanatus* L. subsp. *Vulgaris* (Cucurbitaceae). Mitochondrial DNA B Resour. 1(1):943–944. 10.1080/23802359.2016.1261611 33473686 PMC7800656

[CIT0017] Zhu Q, Zhang M, Cui H, Fan C, Gao P, Wang X, Luan F. 2017. The complete chloroplast genome sequence of the *Citrullus colocynthis* L. (Cucurbitaceae). Mitochondrial DNA B Resour. 2(2):480–482. 10.1080/23802359.2017.1361351 33473871 PMC7800171

