# Supplementary material for: The complete chloroplast genome sequence of Zehneria japonica (Thunb.) H. Y. Liu (Cucurbitaceae), a medicinal plant
Source: Mitochondrial DNA B Resour. 2025 Oct 13;10(11):1037–41. doi: 10.1080/23802359.2025.2571717 (PMC12523466; doi:10.1080/23802359.2025.2571717)
Supplement: Supplementary_material.pdf [file TMDN_A_2571717_SM4538.pdf]

# Supplementary material

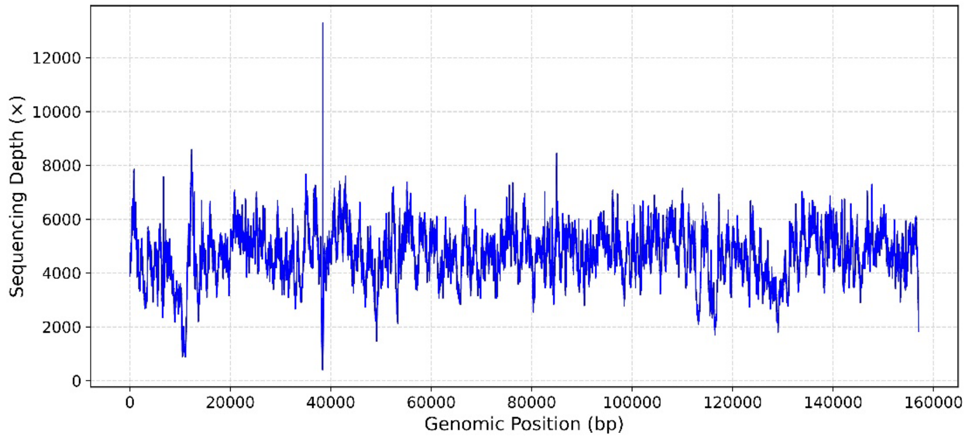


**Figure S1. Sequencing depth and coverage map of the *Zehneria japonica* chloroplast genome.** The sequencing reads coverage and depth of *Zehneria japonica* chloroplast genome is shown in this figure. The minimal, maximal and average read mapping depths were 410×, 13291× and 4705×, respectively. Data analysis and figure was according to method in Ni et al. 2023.

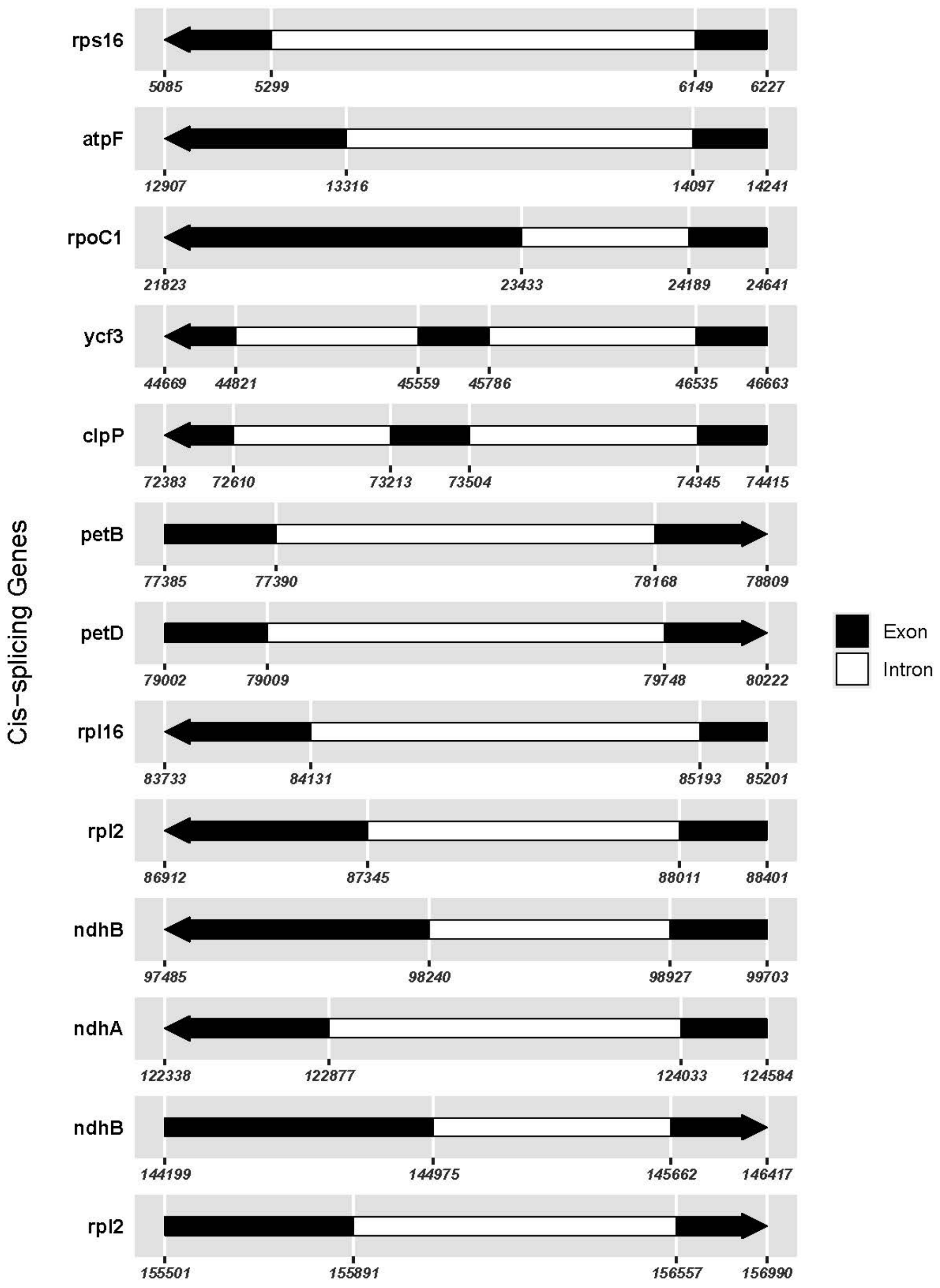


**Figure S2. Schematic map of the cis-splicing genes in the chloroplast genome of *Zehneria japonica*.**

Exons and introns are shown in black and white separately, while arrows indicate the sense direction of transcription. The numbers below each schematic map demonstrate the location in the chloroplast genome. This map was not drawn to scale with CPGView (Liu et al. 2023).

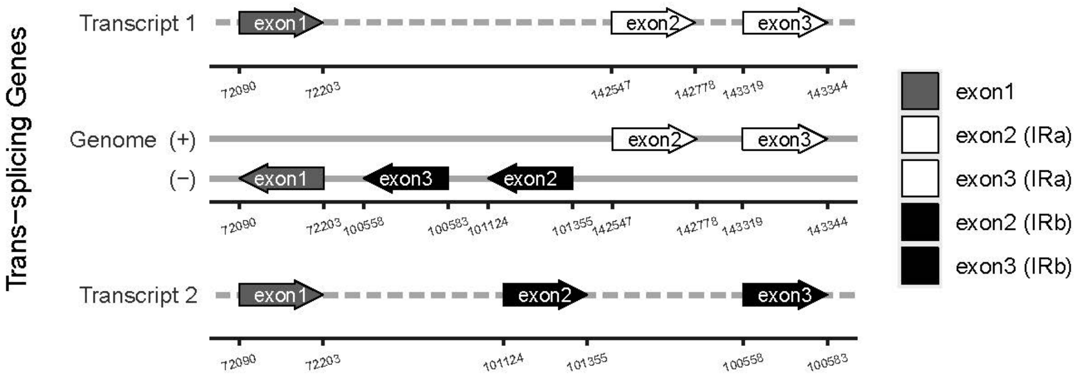


**Figure S3. Schematic map of the trans-splicing gene *rps12* in the chloroplast genome of *Zehneria japonica*.**

The arrows of exons indicate the sense direction of transcription. The numbers below each schematic map represent the location in the chloroplast genome. This map was not drawn to scale with CPGView (Liu et al. 2023).

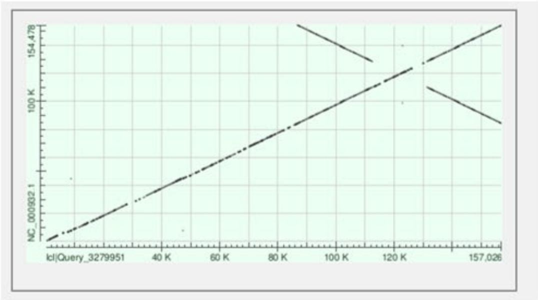

PV819072.1 vs NC_000932.1

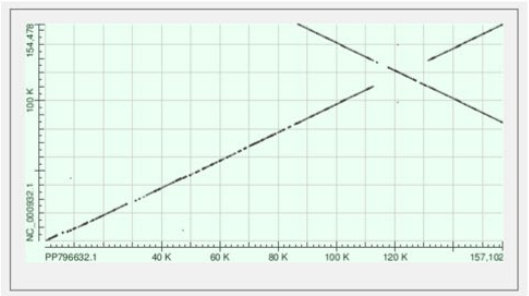

PP796632.1 vs NC_000932.1

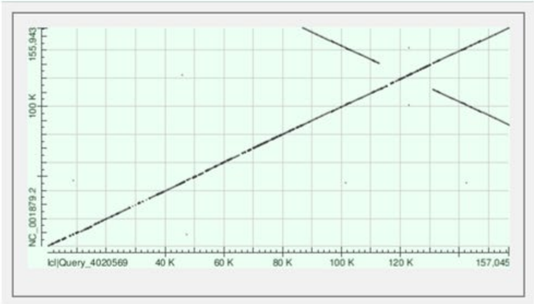

PV819072.1 vs NC_001879.2

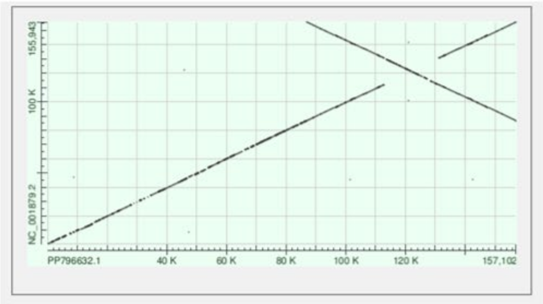

PP796632.1 vs NC_001879.2

**Figure S4. The direction of SSC region in *Z. japonica* is consistent with model plants.** Alignments were performed between *Z. japonica* (PV819072.1) with model plants *Arabidopsis* (NC_000932.1) or Tobacco (NC_001879.2), so it is with *Neoachmandra japonica* (PP796632.1). Alignment was performed with NCBI BLAST.

## References

Liu S, Ni Y, Li J, Zhang X, Yang H, Chen H, liu C. 2023. CPGView: a package for visualizing detailed chloroplast genome structures. Molecular Ecology Resources. 23(3): 694-704. doi: 10.1111/1755-0998.13729.

Ni Y, Li JL, Zhang C, Liu C. 2023. Generating sequencing depth and coverage map for organelle genomes. protocols.io. doi: dx.doi.org/10.17504/protocols.io.4r3l27jkxg1y/v1.
